# Fabrication and characterization of core–shell microparticles containing an aqueous core

**DOI:** 10.1007/s10544-022-00637-9

**Published:** 2022-11-10

**Authors:** Fariba Malekpour Galogahi, Abolfazl Ansari, Adrian J. T. Teo, Haotian Cha, Hongjie An, Nam-Trung Nguyen

**Affiliations:** 1 Queensland Micro- and Nanotechnology Centre, Griffith University, 170 Kessels Road, QLD 4111 Nathan, Australia; 2 School of Engineering and Built Environment, Griffith University, QLD 4111 Nathan, Australia

**Keywords:** Thermal stability, Mechanical stability, Core–shell microparticle, Aqueous core

## Abstract

Core–shell microparticles containing an aqueous core have demonstrated their value for microencapsulation and drug delivery systems. The most important step in generating these uniquely structured microparticles is the formation of droplets and double emulsion. The droplet generator must meet the performance and reliability requirements, including accurate size control with tunability and monodispersity. Herein, we present a facile technique to generate surfactant-free core–shell droplets with an aqueous core in a microfluidic device. We demonstrate that the geometry of the core–shell droplets can be precisely adjusted by the flow rates of the droplet components. As the shell is polymerized after the formation of the core–shell droplets, the resulting solid microparticles ensure the encapsulation of the aqueous core and prevent undesired release. We then study experimentally and theoretically the behaviour of resultant microparticles under heating and compression. The microparticles demonstrate excellent stability under both thermal and mechanical loads. We show that the rupture force can be quantitatively predicted from the shell thickness relative to the outer shell radius. Experimental results and theoretical predictions confirm that the rupture force scales directly with the shell thickness.

**Graphical abstract:**

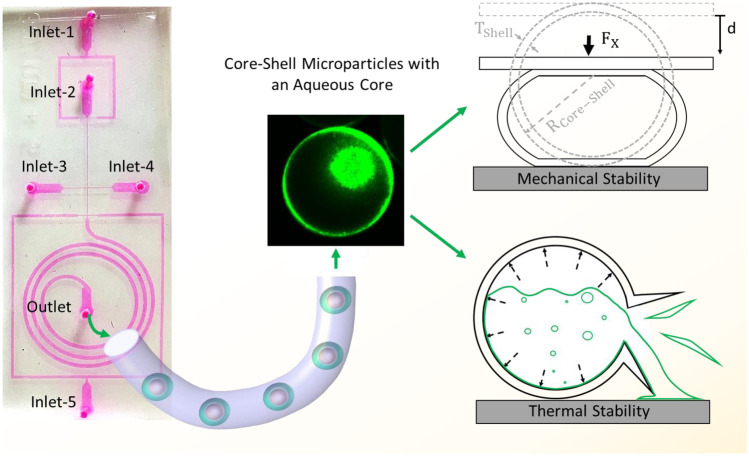

**Supplementary Information:**

The online version contains supplementary material available at 10.1007/s10544-022-00637-9.

## Introduction

Over the last few years, core–shell microparticles with an aqueous core have attracted much attention due to their distinctive chemical and physical properties, as well as broad potential applications. Core–shell microparticles with an aqueous core are promising microencapsulation systems for isolation, protection, and controlled release of encapsulated agents. Microencapsulation is an important process in detergent, food and beverage, water recycling, pharmaceutical, biomedical, and agriculture industries. For instance, the aqueous compartments can entrap water-soluble food additives or sensitive ingredients so that the core–shell particles can serve as a delivery vehicle for particular dietary purposes (Jeyakumari et al. 2016). Core–shell microparticles containing aqueous core can serve as vehicles to deliver water-soluble sex-attractant pheromones to reduce insect populations and protect the crop. The shell protects encapsulated pheromones from degradation and light during storage before final release (Dubey 2009; Mihou et al. 2007). The potential pharmaceutical/ biomedical applications of core–shell microparticles with aqueous core include gene therapy (Jenjob et al. 2020; Davoodi et al. 2017), immobilization of enzymes (Pinto et al. 2019), targeted delivery and controlled release of pharmaceuticals, fabrication of biosensors, single-cell study (Kim et al. 2011), and a 3D matrix (Galogahi et al. 2020). Core–shell particles with a fluorescent aqueous core can be used as indicators in biological and pharmaceutical applications such as cell imaging, cell sorting (Shinde et al. 2015), and sensing (Aslan et al. 2007). The fluorescence intensity of core contents can be used to study the enzymatic activity of the encapsulated cells or their viability during biological processes (Fakhrullin and Minullina 2009). So far, considerable effort has been devoted to applying the core–shell microparticles for practical applications. However, the wide application of these liquid handling platform is hampered by their broad size distribution, lack of control over the structure, content release (Galogahi et al. 2021), and difficulty in scaling up (Galogahi et al. 2020). Another drawback of this platform is the instability and a tendency for coalescence during the production process (Bonnet et al. 2009). Hence, most production approaches have relied extensively on surfactants to prevent the coalescence of droplets. However, surfactants have undesirable side effects, especially for pharmaceutical applications (Hu et al. 2008; Xiong et al. 2008; Xie et al. 2009).

Droplet-based microfluidics is promising tool for rapid, continuous, and scalable production of core–shell droplets with excellent control over their dispersity, size, shape, and structures (Bandara et al. 2015; Sivasamy et al. 2011). Droplet-based microfluidics can combine different lipophilic and hydrophilic layers so that core–shell droplets can simultaneously encapsulate a variety of hydrophobic and hydrophilic ingredients. Flow-focusing is one of the most popular droplet-based microfluidic configuration for the production of core–shell droplets or double emulsion (Shui et al. 2008; Tan et al. 2008). Flow-focusing geometry allows for a wide range of droplet sizes, generation rates, and number of shell layers (Lashkaripour et al. 2019; Teo et al. 2020). However, the relatively large number of effective parameters such as device geometry, fluid properties, and operation conditions have made designing a microfluidic flow-focusing device challenging and has hindered its performance in practical applications (Galogahi et al. 2021a). Various aspect ratios cause the underlying physics and laws to be specific to each study (Lashkaripour et al. 2019). Moreover, droplet-based microfluidic applications usually require modified surface wettability of the microchannels. Patterning the wettability at an acceptable high accuracy and quality has always been challenging (Schneider et al. 2010).

The microfluidics-based core–shell droplets could be further solidified to form a core–shell microparticle with tailored physical and chemical properties for the controlled release of its encapsulated contents. The solidification of the shell prevents leakage during storage, handling, or service and allows triggered release of the encapsulated content through external stimuli, such as mechanical, thermal stress and pH change (Galogahi et al. 2021b; Jeong et al. 2021). Mechanical stimuli-induced release is useful for drug delivery, anticorrosion protection, mechanobiology (Skirtach et al. 2011), intracellular transport of small peptides (Volodkin et al. 2012). Mechanical stimuli such as stretching, shearing, and compression can trigger the release of the content of the microparticles (Rajamanickam et al. 2020). Robust microparticles resist cracking before delivering cargoes to the targeted place, while weak microparticles fail to deliver and reduce encapsulation efficiency (Skirtach et al. 2011). The thermally induced release is useful for applications, where a premature release of contents should be inhibited until reaching targeted temperature. For instance, thermally induced release can help to deliver food additives and ingredients under control upon shell layer melting (Esser-Kahn et al. 2011). For instance, rising soil temperature could trigger the release of encapsulated fertiliser in agriculture applications (Friedman and Mualem 1994; Drake 1988). Temperature change could result in disassembly, decomposition, or melting of polymeric microparticles, ultimately triggering the release (Esser-Kahn et al. 2011). The mechanical and thermal strengths of the shell play a crucial role in rupturing or damaging the microparticles during the production process, maintaining long-term stability and at the same time the sensitivity to mechanical or thermal stress required for the triggered release of core components (Rajamanickam et al. 2016). Identifying the key parameters impacting the mechanical and thermal resistance is critical for the commercialization and practical applications of core–shell microparticles developed in the laboratory. Research has shown that mechanical and thermal strength of the shell significantly depends on the core and shell materials (Galogahi et al. 2021b). For instance, organic materials can cause plasticization of the polymeric shell and reduce its mechanical strength, especially elastic modulus and hardness. Shell thickness and microparticles size can also affect the shell’s elastic modulus (Ahangaran et al. 2019; Zhou et al. 2019). Shell thickness and microparticles size determine the rupture behaviour of microparticles and their resistance against the deformation (Galogahi et al. 2021b). However, it is still unknown how the material properties and geometry quantitatively affect the mechanical and thermal properties of such a complex core–shell structure. Temperature control will allow for a complex manipulation protocol from formation (Murshed et al. 2009) to sorting (Yap et al. 2009), to rupturing the core–shell microparticles.

In this study, we developed surfactant-free core–shell microparticles containing water and fluorescence aqueous core and polymeric shell using core–shell droplets generated as a template in a PDMS-based microfluidic flow-focusing device. We demonstrated how varying flow conditions affect droplet size, shell thickness. In a previous work, we established analytical models for the mechanical behaviour of thick-walled liquid core–shell particle under compression. We validated our models with experiments on core–shell particles with an oil core. However, more experimental information on mechanical behaviour of such a complex structure is required to validate the models. The present study also aims to experimentally and theoretically investigate the behaviour of core–shell microparticles with an aqueous core during compression and heating processes. The experimental results are then compared with those of the analytical models.

## Experiments

### Materials

Silicon wafers were purchased from IBD Technologies Ltd. (Wiltshire, UK). The photoresist SU-8 3050 was obtained from MicroChem Corp (Westborough, USA). PDMS prepolymer and the curing agent (Sylgard 184) were purchased from Dow Corning (Midland, MI, USA). Poly (vinyl alcohol) (PVA) (87–90% hydrolysed, average mol wt. 30,000–70,000) was purchased from Sigma-Aldrich. Trimethylolpropane trimethacrylate (TMPTMA), Ethyl- 4(dimethy-lamino) benzoate, and camphorquinone were purchased from Sigma-Aldrich. Cetyltrimethylammonium bromide (CTAB), glycerol, and fluorescein were purchased from Chem-supply. Deionised water (Millipore) was used to prepare all aqueous solutions.

### Fabrication of the microfluidic device

The PDMS-based microfluidic device was fabricated following the procedure reported in our previous study (Galogahi et al. 2021b). Briefly, a mask was designed using CleWin (WieWeb Software, The Netherlands) to define the patterns and printed on a transparent film. A layer of SU-8 3050 (MicroChem) was spin coated on a 4-inch silicon wafer. This layer was then patterned into the mould of the microchannels through photolithography and hard baking. The microchannel dimensions are 30 µm in constriction width, 100 µm in width, 400 µm in spiral width, and 120 µm in height. The microfluidic device was fabricated with soft lithography approach. A degassed mixture of polydimethylsiloxane (PDMS) base and the curing agent with a ratio of 10:1 was poured onto the SU-8 mould, followed by curing for at least 1 h at 75 °C. The cured PDMS replica was then peeled off. The inlets and outlet were punched using a biopsy puncher. The PDMS device was subsequently bonded onto a glass substrate after treating both PDMS and the glass substrate in an oxygen plasma cleaner (PDC-32G-2, Harrick Plasma).

Selective surface modification of microchannels, necessary for generating core–shell droplets, was achieved via polyvinyl acetate (PVA) deposition. PVA deposition on the PDMS surface results in the long-term and most robust hydrophilicity. PVA solution was prepared by mixing PVA and distilled water using a magnetic stirrer at 100 °C for 3 h. The beaker containing the solution was then weighed, and some water was added to make up for water lost due to evaporation. We manually injected PVA (1 wt%) from the outlet channel to the second junction to maintain the hydrophilicity of this section. Simultaneously, air introduced from the first and second inlets to maintain a hydrophobic section behind the second junction. Air was delivered with a syringe pump at a high flow rate of 400 µL⁄min for 15 min. PVA was then thoroughly removed by blowing air into the channels, and the device was baked at 100 °C for 15 min. The process was repeated three times to achieve an acceptable level of hydrophilicity. Subsequently, we filled the desired channel, which should be hydrophobic, with Aquapel. Aquapel was left inside the microchannels for 5 min. Simultaneously, the air flowed through the hydrophilic channels using a syringe pump at a flow rate of 600 µL⁄min. Finally, Aquapel was entirely removed by blowing air.

### Preparation of core–shell particles

**Fig. 1:**
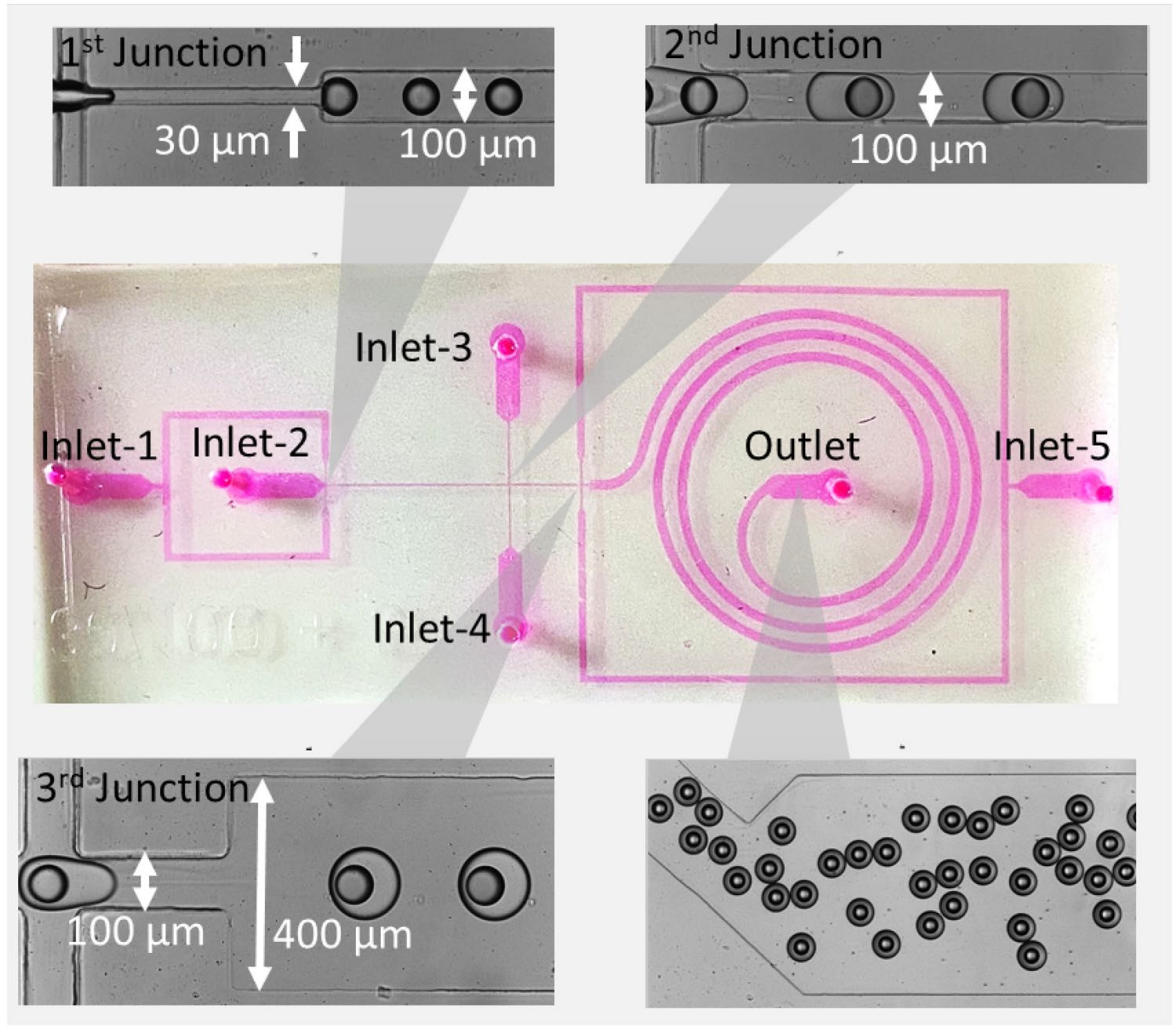
Five-inlet microfluidic device for the generation of core–shell droplets with an aqueous core. Inlet 1: Aqueous core fluid; Inlet 2: TMPTMA; Inlet 3, 4, and 5: Aqueous solution containing 50% v/v glycerol and 0.625 wt % CTAB. Briefly, aqueous core and shell (TMPTMA) fluids were injected from the first and second inlets, resulting in the core droplets. The continuous phase was introduced from the third and the fourth inlets, resulting in subsequent formation of core–shell droplets. The spacer fluid was inserted at the third junction to prevent accidental merging of core–shell droplets. The shells were then partially cross-linked by exposure to UV while passing through the spiral channel. The core–shell particles were collected at the outlet

The flow-focusing microfluidic device consisted of five inlets and one outlet, as schematically shown in Fig. 1. The inlet fluids were all delivered into the microfluidic device using a syringe pump (NEM-B101-03 A, CETONI GmbH, Germany) at controlled flow rates. The core and shell fluids enter from the first and the second inlets, respectively. The third and the fourth inlets introduced the continuous phase. The final inlet inserted a spacer fluid ${Q}_{s}$ at a flow rate of 200 µL/h to prevent accidental merging of core–shell droplets. The flow rates of the core fluid ${Q}_{\mathrm{C}}$ and the shell fluid ${Q}_{\mathrm{sh}}$ were varied to investigate their impact of flow conditions on the formation of core–shell droplets. First, the core flow ${Q}_{\mathrm{C}}$ was changed from 10 to 40 µL/h, while the flow rates of the shell fluid ${Q}_{\mathrm{sh}}$ and continuous fluid ${Q}_{\mathrm{co}}$ at first and the second cross junctions were kept at 120 and 200 and 200 µL/h, respectively. Next, the flow rate of the shell fluid ${Q}_{\mathrm{sh}}$ was changed from 80 to 160 µL/h, while the flow rates of core fluid ${Q}_{\mathrm{c}}$ and continuous fluid ${Q}_{Co}$ at the first and second junctions were kept at 20 and 200 and 200 µL/h, respectively. For the generation of core–shell droplets with an aqueous core, the core and the shell phase were composed of distilled water and TMPTMA, respectively. The TMPTMA was formulated by mixing 0.06 g of ethyl- 4(dimethylamino)benzoate, 0.05 g of camphorquinone, and 10 g of TMPTMA using mechanical stirring at 600 rpm for 1 h. For the generation of the fluorescent cores, the core phase consisted of 0.5% wt fluorescein. The continuous phase and the spacer fluid were the aqueous solution, constituted of 50% v/v glycerol and 0.625% wt CTAB. Finally, the generated core–shell droplets left the device through the outlet tubing. The spiral channel and outlet tubing were exposed to a blue light source (110–240 V, 24 W) to partially polymerise the shell and to prevent accidental aggregation while the droplets are passing inside the channel and tubing. The spiral geometry with a relatively large width of 400 µm also prevents the core from escaping the shell layer after the formation of the core–shell droplets by decreasing the shear stress and increasing the shell curing time. The partially cross-linked particles were subsequently exposed further to the blue light for 30 min after collection to be fully cross-linked. After the exposure, the generated core–shell particles were rinsed with distilled water three times to remove glycerol and CTAB. However, past studies have indicated no severe toxicity problems if CTAB concentration is kept at a minimum (Wang et al. 2008; Singh et al. 2000). Nevertheless, we used the Raman spectroscopic technique to discover the possible presence of CTAB residues on the surface of particles. We then left the core–shell particles at room temperature for five days to be completely dried.

The formation process of core–shell droplets was observed in real-time and recorded using a high-speed CCD camera (Phantom Miro3, Vision Research) attached to an inverted microscope (Nikon, Eclipse Ti). We used ImageJ to analyse video frames captured and to measure the size of the core–shell droplets. The surface and cross-sectional microstructures of core–shell particles were studied using scanning electron microscopy (SEM) operating at an acceleration voltage of 1 kV. To obtain the cross-sections of fractured particles, they were sliced manually with a scalpel blade. The morphology of core–shell particles was characterised by optical microscopy using an inverted microscope (Eclipse Ti2, Nikon).

### Mechanical behaviour of core–shell microparticles

We conducted further experiments to verify the theory of compression of fluid-filled elastic shells previously developed. Figure S2(a) (supporting Information) shows the experimental setup designed and built for the compression test. A single-axis translation stage with the 150-811ST micrometre drive was positioned perpendicular to the pan of an analytical balance (Sartorius Entris1241-1S, Sartorius Co., Germany). The microparticles were compressed using a right-angle mounting adapter mounted on the translation stage as it was steered downward. The side view of the compression process was observed and recorded continuously using a video camera (XimeaxiQ-USB3 MQ013CG-ON, Edmund Optics Co.) with a 0.7–4.5X Zoom Imaging Lens (VZM450, Edmund Optics Co.). During all the experiments, the forces being imposed on the microparticles were measured using the analytical balance. Finally, the displacements created by applying force due to the compression of the microcapsules were measured using ImageJ. Each experiment was repeated three times to achieve the average values.

### Thermal behaviour of core–shell microparticles

The thermal stability of the core–shell particles was evaluated with a customized setup, Fig. S2(b) (supporting Information). Core–shell particles were gently transferred on a glass slide which was located on a controlled hot plate. Temperature scans started by holding the particles for 10 min at 25 °C followed by heating at a particular increment of 10 °C until the shell rupture was initiated. The core–shell particles were maintained at each temperature for 10 min to achieve a good isothermal condition across the particles. During the heating process, a video camera (XimeaxiQ-USB3 MQ013CG-ON) with 0.7–4.5X Lens (VZM450, Edmund Optics Co.) placed on top of the sample simultaneously recorded the data. Each experiment was repeated three times to achieve the average values of the critical rupture temperature.

## Result and discussion

### Effects of fluids flow rates on core–shell emulsion

**Fig. 2:**
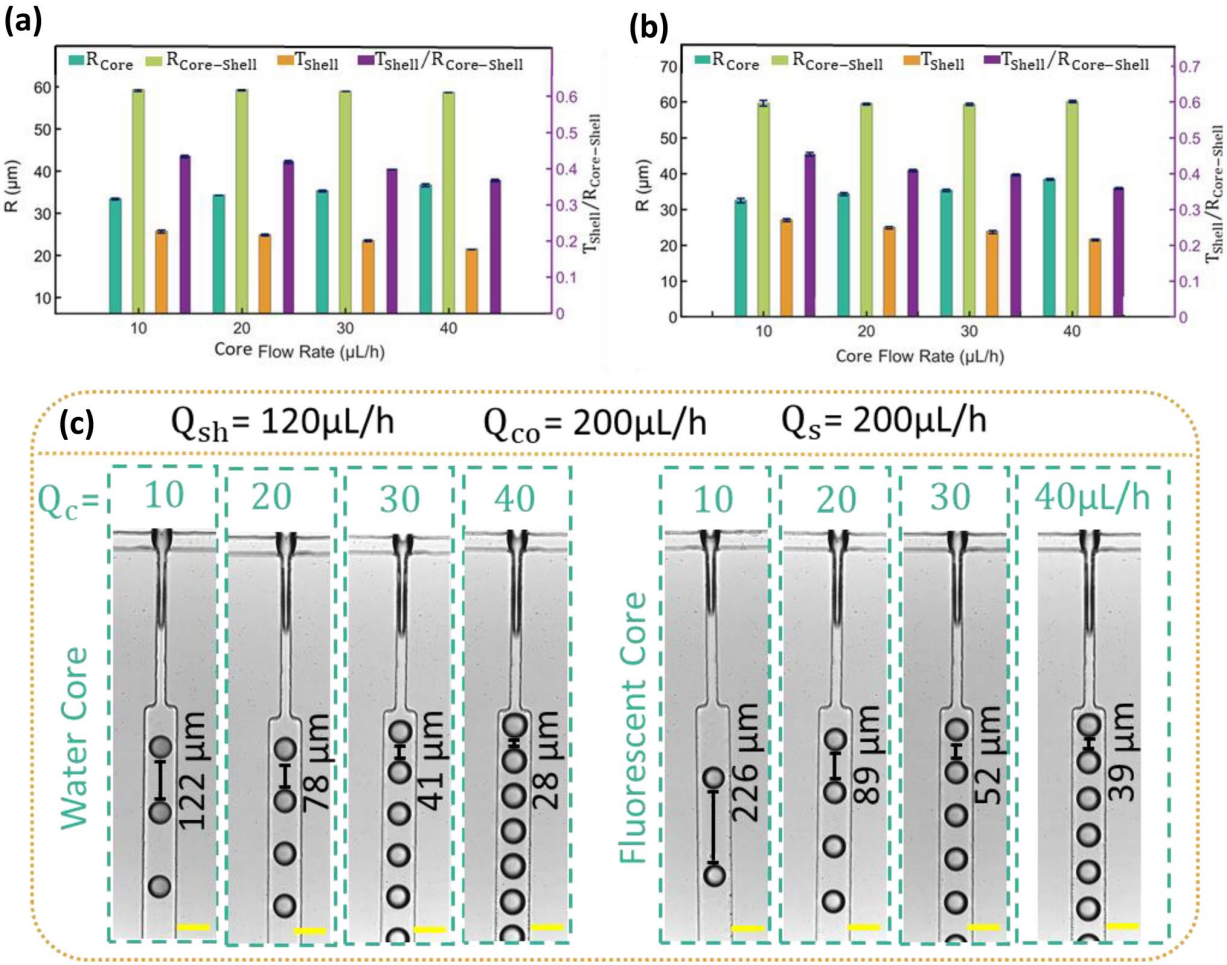
Final core–shell droplet with **a** a water core and **b** a fluorescent core geometry plotted against the core flow rate ${Q}_{c}$; **c** Images of the first junction showing the formation of water core and fluorescent core droplets at a different core flow rate. Scale bars depict 100 μm

**Fig. 3:**
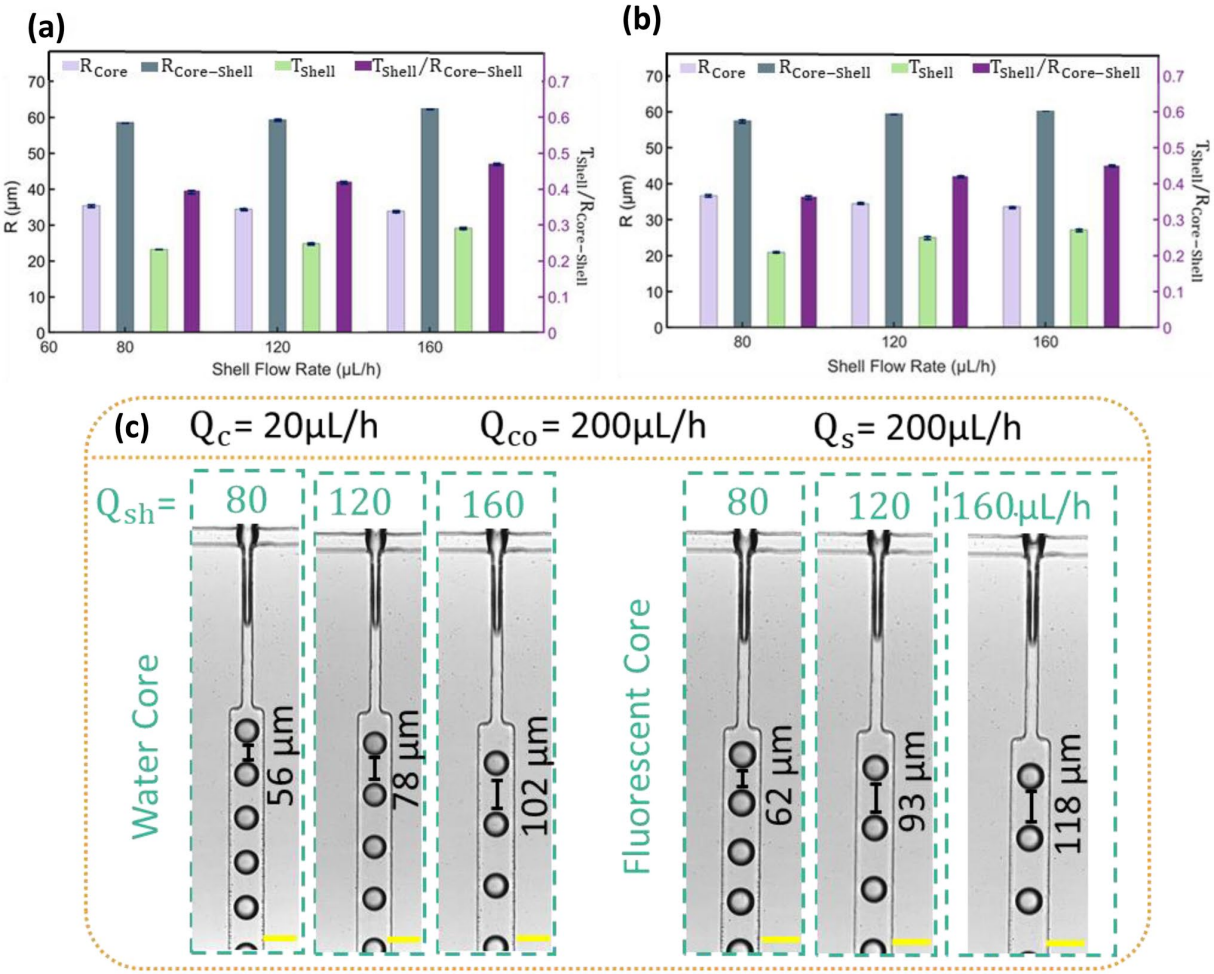
Core–shell droplet with **a** a water core and **b** a fluorescent core geometry plotted against the shell flow rate ${Q}_{sh}$; **c** Images of the first junction showing the formation of water core and fluorescent core droplets at a different shell flow rate. Scale bars depict 100 μm

Figure 2 shows the effect of variation in water and fluorescent solution flow rate ${Q}_{\mathrm{C}}$ on the core ${R}_{\mathrm{Core}}$ and core–shell ${R}_{\mathrm{Core}-\mathrm{Shell}}$ droplet radius and the shell thickness ${T}_{\mathrm{Shell}}$. Figure 3(a) indicates that increasing the core flow rates ${Q}_{\mathrm{C}}$ from 10 to 40 µL/h results in larger water and fluorescent core droplets, while the separation distance between the droplets decreases. In contrast to the core droplets, the radius of shell droplets ${R}_{\mathrm{Core}-\mathrm{Shell}}$ is independent of the core flow rates and remains at about 59 µm for the different core flow rates. Consequently, with the growing size of core droplets, the thickness of the shell ${T}_{\mathrm{Shell}}$ reduces. The droplet formation in flow-focusing geometry relies on three main forces: the interfacial force withstanding the break-up, the shear force acting on the droplet generation by the continuous phase, and hydrostatic pressure. In the dripping regime, the interfacial force initially dominates, and the interface starts to expand in the radial and axial direction towards the channel. Eventually, after blocking the channel of the continuous phase and increasing the pressure, the shear force overcomes the interfacial tension leading to droplet breakup. Increasing the core flow rate amplifies the expansion of the interface, resulting in larger droplets. Figure 2 also shows that adding fluorescent dye to the core has a negligible impact on the core and the size of the core–shell droplet, while it significantly affects the separation distance between core droplets, resulting in larger spacing gap. For example, the addition of fluorescent dye dramatically increases the separation distance to 80% in the flow rate of 10 µL/h. For a higher flow rate, this impact decreases to 38%.

Figure 3 compares the core ${R}_{\mathrm{Core}}$ a and core–shell ${R}_{\mathrm{Core}-\mathrm{Shell}}$ a droplet size and the shell thickness ${T}_{\mathrm{Shell}}$ at increasing shell flow rate ${Q}_{\mathrm{sh}}$ while the core ${Q}_{\mathrm{C}}$ and continuous ${Q}_{\mathrm{Co}}$ and spacer fluid ${Q}_{\mathrm{s}}$ flow rates are kept constant at 20, 200, 200 and 200 µL/h, respectively. Figure 3 illustrates that increasing shell flow rate resulted in smaller water and fluorescent core droplets. While the radius of core–shell droplets ${R}_{\mathrm{Core}-\mathrm{Shell}}$ a raised by almost 7% due to a two-fold increase in the shell flow rate. Accordingly, a significant increase in the thickness of the shell can be observed. Comparing Fig. 3(a, b) indicates that the addition of fluorescent dye to core had almost no effect on core and core–shell droplets size. However, the addition of fluorescent dye increased the separation distance between the core droplets. It is also apparent that the increase of shell flow rate increases the separation distance between the core droplets.

**Fig. 4:**
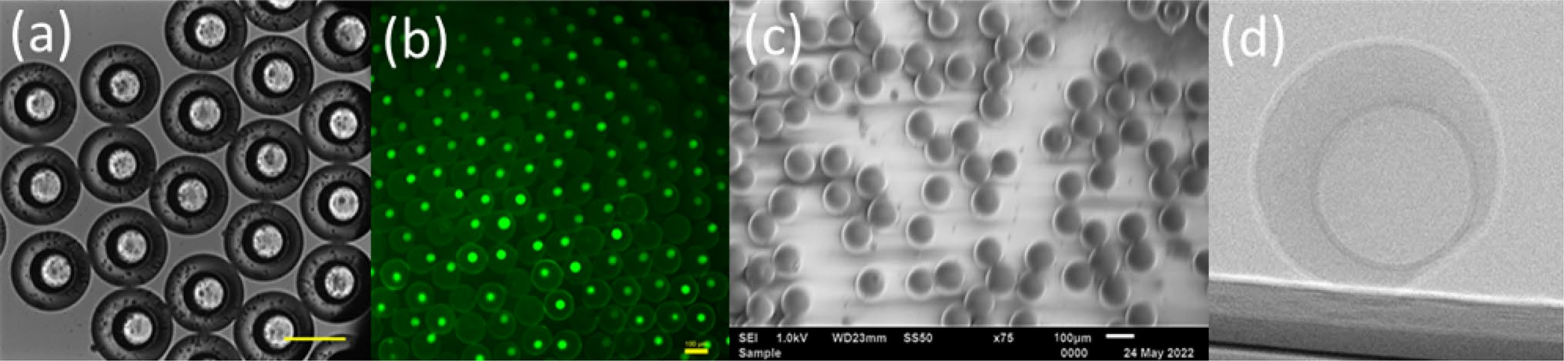
Microscopic images of **a** core–shell droplets with water core after collection and **b** core–shell droplets with fluorescent core. **c** SEM image of solid core–shell microparticles after curing and drying. **d** X-ray image of a core–shell particle. Scale bars depict 100 μm

Figure 4(a, b), show representative microscopic images of these core–shell droplets after collection. The images reveal a spherical, smooth, and homogeneous shell encapsulating the aqueous core. The morphology and microstructure of solid microparticles were characterised by scanning electron microscopy (SEM). We found that individual microparticles have regular morphology, smooth surface, Fig. 4(c). Almost all core–shell microparticles maintained their spherical shape after curing and drying.

### Mechanical behaviour of core–shell microparticles

**Fig. 5:**
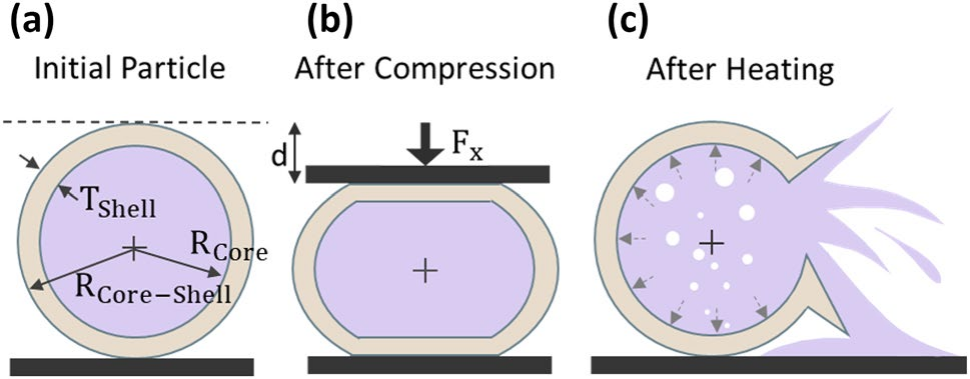
Schematic figures showing **a** the initial core–shell particle with water core, **b** after compression between two parallel plates, and **c** after heating up to critical rupture temperature

**Fig. 6:**
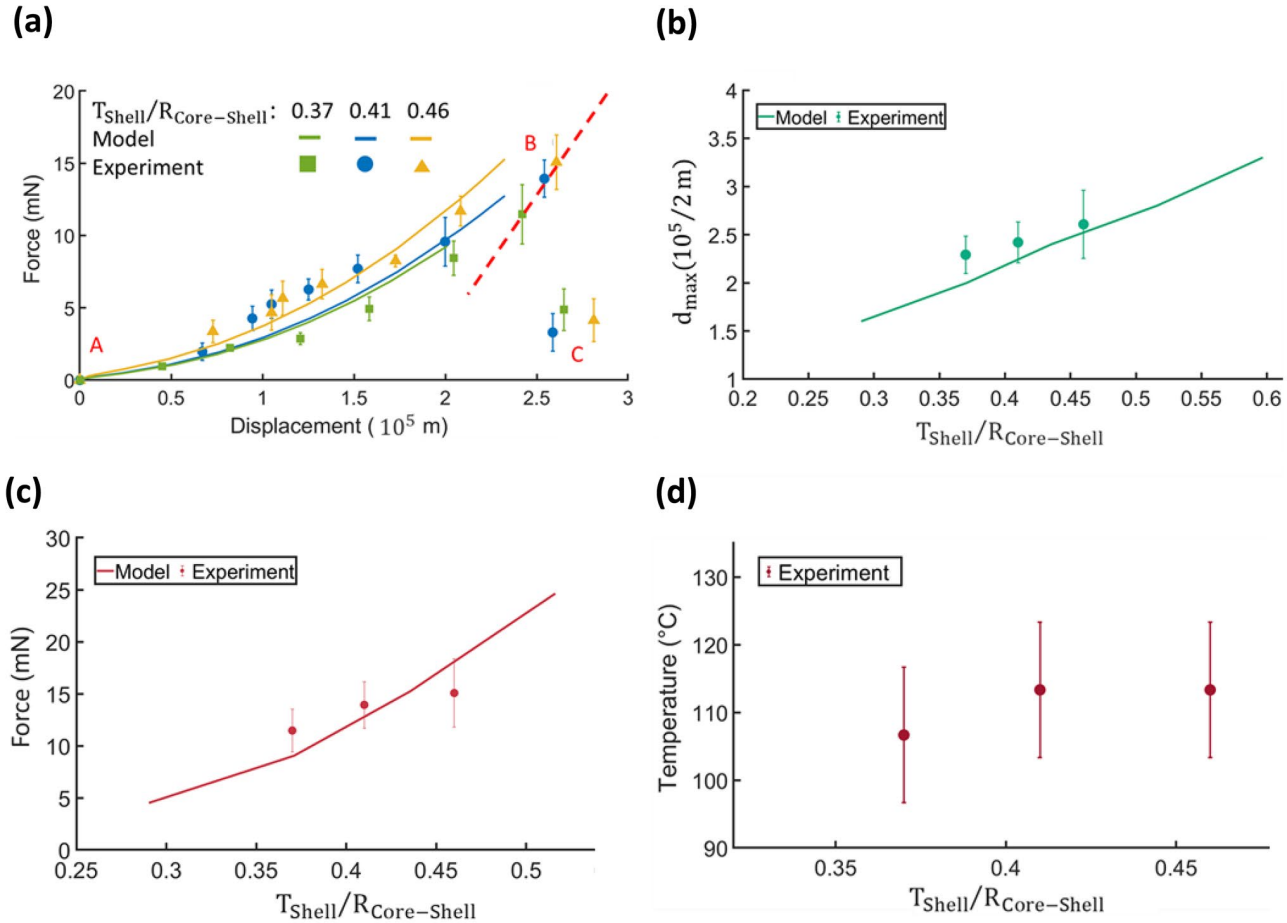
**a** Force versus displacement curve when single microparticle was compressed to the rupture point; **b** The critical rupture displacement ${d}_{max}$ versus ${T}_{Shell}/{R}_{Core-Shell}$ at the rupture point; **c** Rupture force versus ${T}_{Shell}/{R}_{Core-Shell}$; **d** Critical rupture temperature versus ${T}_{Shell}/{R}_{Core-Shell}$

This section discusses with the deformation behaviour of core–shell particles with water core under compression. We considered a thick-walled spherical shell of outer radius ${R}_{\mathrm{Core}-\mathrm{Shell}}$ and thickness ${T}_{\mathrm{Shell}}$ in contact with two parallel rigid plates, Fig. 5(a, b). During the compression process, the initial response of the shell was flattening against the plates, followed by sudden rupture. Pauchard and Rica (1998) were pioneers in developing the theory of compression of elastic shells. The authors experimentally deduced the elastic energy stored in the hollow shell due to the displacement corresponding to the deformation. In our previous work, we investigated whether Pauchard’s model could describe the deformation behaviour of the elastic shell filled with an incompressible oil during compression. Herein we explore whether the model is still valid in the case of a particle filled with water. Figure 6(a) shows the response of microparticles with a water core to the application of force predicted using the axisymmetric model (Supporting Information, Eq. (3)). The responses were then compared with experimentally measured data. We evaluated and compared the mechanical properties of three different core–shell particles with water core. The ratios of the shell thickness to outer radius ${T}_{\mathrm{Shell}}/{R}_{\mathrm{Core}-\mathrm{Shell}}$ of microparticles under investigation were 0.37, 0.41 and 0.46, respectively. Data were fitted to Eq. (3) (Supporting Information), which generated fitting parameters *A* = 0.05 and *B* = 0.06. In Fig. 6(a), point A in the curve represents the start of the microparticles being touched by the top plate. Curve A-B corresponds to the compression and deformation of microparticles being held between two plates. The microparticles were then ruptured at point B and the force instantaneously dropped due to the release of the water core, represented by point C. The deviation of experimental results from the theoretical predictions might be due to the lack of concentricity. Figure 6(b) shows the critical rupture displacement ${d}_{\mathrm{max}}$ as a function of shell thickness to outer radius ${T}_{\mathrm{Shell}}/{R}_{\mathrm{Core}-\mathrm{Shell}}$ obtained by equalizing Eqs. (1) and (2) (Supporting Information) with a C value of 0.048. The Fig. 6(b) indicates that critical displacement ${d}_{\mathrm{max}}$ of the particle with higher ${T}_{\mathrm{Shell}}/{R}_{\mathrm{Core}-\mathrm{Shell}}$ is greater than that with lower ${T}_{\mathrm{Shell}}/{R}_{\mathrm{Core}-\mathrm{Shell}}$, because the particle with higher ${T}_{\mathrm{Shell}}/{R}_{\mathrm{Core}-\mathrm{Shell}}$ ratio had a much thicker shell. The core–shell microparticles burst when they were deformed by almost 60%. The generated critical displacement ${d}_{max}$ data was then used to determine the corresponding rupture force using Eq. (3) (Supporting Information), Fig. 6(c). Figure 6(c) displays that by increasing the shell thickness to outer radius ${T}_{\mathrm{Shell}}/{R}_{\mathrm{Core}-\mathrm{Shell}}$, the resistance of particles to deformation increases, consistent with the experimental results. The rupture force of core–shell particles with an ${T}_{\mathrm{Shell}}/{R}_{\mathrm{Core}-\mathrm{Shell}}$ ratio of 0.46 was approximately 30% greater than that with an ${T}_{\mathrm{Shell}}/{R}_{\mathrm{Core}-\mathrm{Shell}}$ ratio of 0.37.

### Thermal behaviour of core–shell microparticles

This section investigates the thermal properties of core–shell particles with water core. We experimentally studied the behaviour of three core–shell particles with the shell thickness to the outer radius ${T}_{\mathrm{Shell}}/{R}_{\mathrm{Core}-\mathrm{Shell}}$ ratios of 0.37, 0.41, and 0.46. The microcapsules were heated from 25 °C with a temperature increment of 10 °C until bursting and releasing the core content. The results indicated that the microparticles remained approximately unchanged and stable up to the critical rupture temperature. All microparticles burst and released the water core at a temperature more than 100 °C, beyond the boiling temperature under atmospheric condition. The average critical rupture temperatures of microparticles with ${T}_{Shell}/{R}_{Core-Shell}$ ratios of 0.37, 0.41, and 0.46 were 106 °C, 113 °C, and 113 °C, respectively. The broken release happened when water pressure inside the microparticles increased beyond the shell’s yield point under heating (Fig. 5(c)). We considered that the pressures build in an isochoric process as the internal volume of particles remains constant throughout the heating process. In these closed systems of constant volume, the isochoric process occurs in the undercooled compressed liquid phase. Heating increases the temperature and the corresponding pressure. The corresponding experimental rupture pressure can be estimated as the saturation pressure at the given temperature from the saturated water tables, e. g. 1.2714 bar and 1.7682 bar for 106 °C and 113 °C, respectively (Rajput 2009). The average rupture pressure ${P}_{i}$ corresponds to the maximum radial normal stress inside the shell (Eq. 5, Supporting Information). In agreement with (5), the experimental results indicated that the rupture temperature of the microparticles is independent of ${T}_{Shell}/{R}_{Core-Shell}$ ratio (Fig. 6(d)). Based on the experimental results the rupture temperature is almost unaffected by the proprieties of the shell layer. The perversion of microparticle with ${T}_{Shell}/{R}_{Core-Shell}$ ratio of 0.37 might be due to the asymmetry of the aqueous core in the shell, Fig. 4(d). As shown in microscopic (Fig. 4(b)) and x-ray images (Fig. 4(d)), the location of cores and the shell shape may vary. Some of the cores are located closer to the outer surface. The nonuniformity in the shell thickness results in nonuniform normal stress to the shell layer. Consequently, the shell begins to buckle from the weak spot under pressure. The shell from the thin-walled part ruptures more easily than the thicker part and earlier than the average rupture temperature (Paulose and Nelson 2013; Datta et al. 2012; Zhang et al. 2019).

## Conclusion

We developed a facile microfluidic approach for producing uniform core–shell microparticles with an aqueous core in a flow-focusing geometry. The approach involves precise and simple control of the hydrophilicity and hydrophobicity of microchannels and without surfactant in both core and shell layers. The spiral geometry with the increased width of 400 µm in the present work prevents the core to escape from the shell layer by decreasing the shear stress and increasing the droplet’s curing time. Low toxicity and biocompatibility of aqueous core and shell polymer make our microparticles highly practical and attractive for encapsulating various biologically active components. The further advantage of our microparticles is the simultaneous potential encapsulation of hydrophilic and hydrophobic molecules into the core and shell layer, respectively. We subsequently studied the mechanical and thermal responses of resultant microparticles undergoing compression and heating. The results revealed that critical temperature and rupture forces depended strongly upon the shell thickness to the outer radius. The critical temperature, the force required to rupture microparticles and rupture displacement increased with the shell thickness to the outer radius. The predictions of our models were in excellent agreement with the experimental data across all three geometries. Our study revealed that the high mechanical and thermal stability of microparticles with water core might have potential for applications requiring high temperature and mechanical stability. For instance, these core–shell particles could be applied as a bioreactor to condense and encapsulate deoxyribonucleic acid (DNA). The shell can resist the relatively high temperature of thermal cycling of polymerase chain reaction. The transparency of shell material allows for monitoring and evaluating the fluorescence intensity of amplified DNA. Our study has also shown that the shell has the potential to protect DNA against degradation due to high shear forces.

## Supplementary Information

Below is the link to the electronic supplementary material.

Supplementary file1 (DOCX 1143 KB)

## References

[CR1] Ahangaran F, Navarchian AH (2019). F. Picchioni.

[CR2] K. Aslan, M. Wu, J.R. Lakowicz, C.D. Geddes (2007). 10.1021/ja0680820 10.1021/ja0680820 PMC6848855 17283994

[CR3] Bandara T, Nguyen NT (2015). G. Rosengarten.

[CR4] Bonnet M, Cansell M, Berkaoui A, Ropers M-H, Anton M (2009). F. Leal-Calderon.

[CR5] S.S. Datta, S.-H. Kim, J. Paulose, A. Abbaspourrad, D.R. Nelson, D.A. Weitz (2012). 10.1103/PhysRevLett.109.134302 10.1103/PhysRevLett.109.134302 23030092

[CR6] P. Davoodi, M.P. Srinivasan, C.H. Wang (2017). 10.1039/c7tb00481h 10.1039/c7tb00481h 32264215

[CR7] R. Dubey (2009). 10.14429/dsj.59.1489

[CR8] C.F. Drake, Controlled delivery agricultural capsule and method of making. (1988)

[CR9] A.P. Esser-Kahn, S.A. Odom, N.R. Sottos, S.R. White, J.S. Moore (2011). 10.1021/ma201014n

[CR10] R.F. Fakhrullin, R.T. Minullina (2009). 10.1021/la901395z 10.1021/la901395z 19505153

[CR11] Friedman S (1994). Y. Mualem.

[CR12] F.M. Galogahi, Y. Zhu, H. An, N.-T. Nguyen (2020). 10.1016/j.jsamd.2020.09.001

[CR13] F.M. Galogahi, Y. Zhu, H. An, N.-T. Nguyen (2021a). 10.1007/s10404-021-02483-2

[CR14] F.M. Galogahi, H. An, Y. Zhu, N.-T. Nguyen (2021b). 10.1016/j.molliq.2021.117726

[CR15] S.-H. Hu, D.-M. Liu, W.-L. Tung, C.-F. Liao, S.-Y. Chen (2008). 10.1002/adfm.200800428

[CR16] Jenjob R, Phakkeeree T (2020). D. Crespy.

[CR17] H.S. Jeong, E. Kim, C. Nam, Y. Choi, Y.J. Lee, D.A. Weitz, H. Lee, C.H. Choi (2021). 10.1002/adfm.202009553

[CR18] Jeyakumari A, Zynudheen A (2016). U. Parvathy.

[CR19] C. Kim, S. Chung, Y.E. Kim, K.S. Lee, S.H. Lee, K.W. Oh, J.Y. Kang (2011). 10.1039/c0lc00036a

[CR20] Lashkaripour A, Rodriguez C, Ortiz L (2019). D. Densmore.

[CR21] Mihou A, Michaelakis A, Krokos F, Mazomenos B (2007). E. Couladouros.

[CR22] Murshed S, Tan SH, Nguyen NT, Wong TN (2009). L. Yobas.

[CR23] Pauchard L (1998). S. Rica.

[CR24] M.C.C. Pinto, N.L. de Souza e Castro, E.P. Cipolatti, R. Fernandez‐Lafuente, E.A. Manoel, D.M.G. Freire, J.C. Pinto (2019). 10.1002/mren.201800055

[CR25] J. Paulose, D.R. Nelson (2013). 10.1039/c3sm50719j

[CR26] Rajamanickam R, Baek S, Gwon K, Hwang Y, Shin K (2016). G. Tae.

[CR27] Rajamanickam R, Kwon K (2020). G. Tae.

[CR28] R. Rajput, Engineering thermodynamics: A computer approach (si units version), 3rd edn. (Jones & Bartlett Publishers, 2009)

[CR29] Schneider MH, Willaime H, Tran Y, Rezgui F (2010). P. Tabeling.

[CR30] S. Shinde, Z. El-Schich, A. Malakpour, W. Wan, N. Dizeyi, R. Mohammadi, K. Rurack, A. Gjorloff Wingren, B. Sellergren (2015). 10.1021/jacs.5b08482 10.1021/jacs.5b08482 26414878

[CR31] L. Shui, F. Mugele, A. van den Berg, J.C. Eijkel (2008). 10.1063/1.3000624

[CR32] M. Singh, M. Briones, G. Ott, D. O'Hagan (2000) 10.1073/pnas.97.2.811 PMC15413 10639162

[CR33] J. Sivasamy, T.-N. Wong, N.-T. Nguyen, L.T.-H. Kao (2011). 10.1007/s10404-011-0767-8

[CR34] Skirtach AG, Yashchenok AM (2011). H. Mohwald.

[CR35] Tan S-H, Murshed SS, Nguyen N-T, Wong TN (2008). L. Yobas.

[CR36] A.J. Teo, F. Malekpour-galogahi, K.R. Sreejith, T. Takei, N.-T. Nguyen (2020). 10.1063/5.0004736

[CR37] Volodkin D, Skirtach A (2012). H. Möhwald.

[CR38] S. Wang, W. Lu, O. Tovmachenko, U.S. Rai, H. Yu, P.C. Ray (2008) 10.1016/j.cplett.2008.08.039 PMC3780398 24068836

[CR39] M.-Y. Xie, L. Yu, H. He, X.-F. Yu (2009). 10.1016/j.jssc.2008.12.011

[CR40] H.M. Xiong, X.Y. Guan, L.H. Jin, W.W. Shen, H.J. Lu, Y.Y. Xia (2008). 10.1002/anie.200705942

[CR41] Y.-F. Yap, S.-H. Tan, N.-T. Nguyen, S.S. Murshed, T.-N. Wong, L. Yobas (2009). 10.1088/0022-3727/42/6/065503

[CR42] W. Zhang, L. Qu, H. Pei, Z. Qin, J. Didier, Z. Wu, F. Bobe, D.E. Ingber, D.A. Weitz (2019). 10.1002/smll.201903087 10.1002/smll.201903087 31448553

[CR43] Zhou X, Li W, Zhu L, Ye H (2019). H. Liu.

