# Supplementary material for: Fabrication and characterization of core–shell microparticles containing an aqueous core
Source: Biomed Microdevices. 2022 Nov 10;24(4):40. doi: 10.1007/s10544-022-00637-9 (PMC9649509; doi:10.1007/s10544-022-00637-9)
Supplement: Supplementary file 1 — Supplementary file1 (DOCX 1143 KB) [file 10544_2022_637_MOESM1_ESM.docx]

Supplementary Information

Fabrication and Mechanical and Thermal Characterization

of Core-Shell Microparticles Containing an Aqueous Core

Fariba Malekpour Galogahi^1^, Abolfazl Ansari^2^, Adrian JT Teo^1^, Haotian Cha^1^, Hongjie An^1^, Nam-Trung Nguyen^1^

^1^Queensland Micro- and Nanotechnology Centre, Griffith University, 170 Kessels Road, 4111, Queensland, Australia

^2^School of Engineering and Built Environment, Griffith University, Brisbane, QLD 4111, Australia

Corresponding Author: Nam-Trung Nguyen − Queensland Micro and Nanotechnology Centre, Griffith University, Brisbane, Queensland 4111, Australia; orcid.org/0000-0003-3626-5361; Phone: +61- (07)-3735-3921; Email: [nam-trung.nguyen@griffith.edu.au](mailto:nam-trung.nguyen@griffith.edu.au); Fax: +61-(07)-3735-80211

S1. Raman spectra of Core-shell Particles and CTAB

The figure shows the signals of methylene scissoring vibration and asymmetric bending mode of the head [N(CH3)3] methyl group of pure CTAB between 1460 and 1485 ${cm}^{-1}$. The appearance of no peaks of CTAB in core-shell particles indicates no significant interaction or corporation between CTAB and particles.


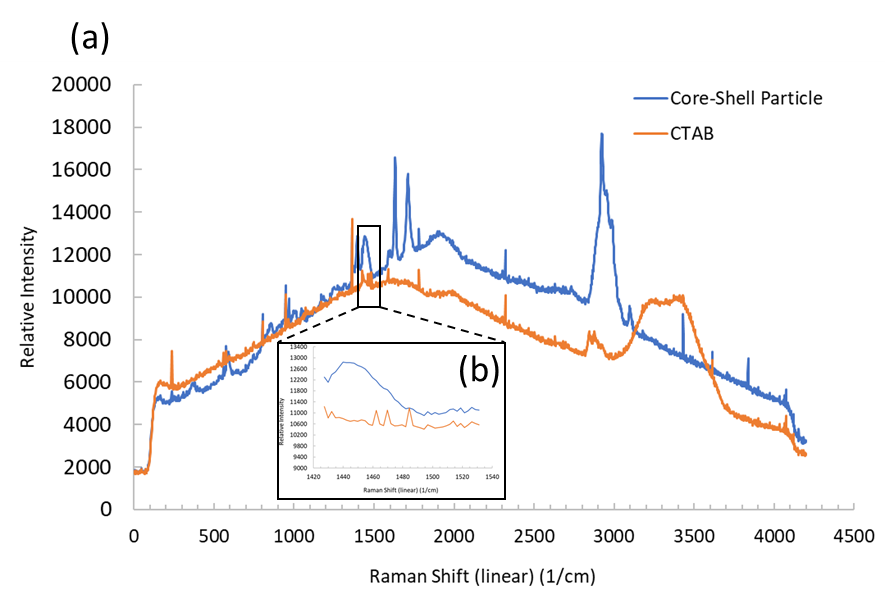


1. UV-Raman spectra of core-shell particles and pure CTAB; (b) methylene scissoring vibration and asymmetric bending mode of the head [N(CH3)3] methyl group of CTAB.

S2. Overview of the experimental setup used for a) compression and b) heating tests:


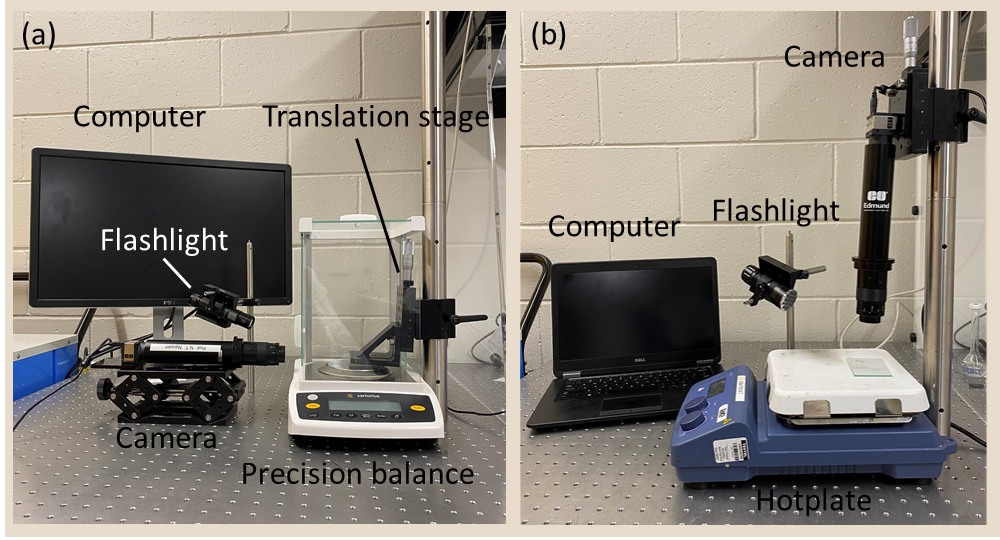


S3. Mechanical behaviour of core-shell microparticles

We considered a thick-walled spherical shell of thickness $T_{Shell}$ in contact with two parallel rigid plates. First, the shell's top and bottom contact surface flattened against the flat plates under low force. Increasing the force beyond the elastic limit led to creating a circular fold in the flattened regions. The elastic energy of the flattened configuration can be calculated by the following equation:

$U_{1}=A\frac{1}{4}\frac{E{T_{Shell}}^{5/2}}{R_{Core-Shell}}{(\frac{d}{2})}^{3/2}+B\frac{ET_{Shell}}{R_{Core-Shell}}{(\frac{d}{2})}^{3}$ (1) where *d* is total deflection of the shell from the top and bottom, *E* is the elastic modulus, and $R_{Core-Shell}$ is outer radius of the shell. *A* and *B* are dimensionless parameters depending only on the Poisson ratio. The first term is contributed to an axisymmetric circular fold of the shell, and the second arises from the flattening shell cap into a flat plane. The elastic energy of the folded configuration also comprises two contributions, which is

$U_{2}=A\frac{E{T_{Shell}}^{5/2}}{R_{Core-Shell}}{(\frac{d}{2})}^{3/2}+C\frac{E{T_{Shell}}^{3}}{R_{Core-Shell}}(\frac{d}{2})$ (2)

where C is constant depending only on the Poisson ratio. The first term results from circularly folding the contact surface inwards, and the second is due to the inversion of the cap. The corresponding displacement change leads to an elastic energy term proportional to ${(\frac{d}{2})}^{3}$. This cubic dependence dominates for large deformations and the force required to make such a deformation increases very quickly, thus, it is not surprising that the system makes a transition to a lower energy configuration of $U_{2}$. The critical deflection $d_{max}$ happen when the elastic energy of the flattened $U_{1}$ and folded configuration $U_{2}$ is equal. The forces acting on the shell during compression test can be given by the first derivative of equations (1):

$F_{x}=A\frac{3}{8}\frac{E{T_{Shell}}^{5/2}}{R_{Core-Shell}}{(\frac{d}{2})}^{1/2}+3B\frac{ET_{Shell}}{R_{Core-Shell}}{(\frac{d}{2})}^{2}$ (3)

S4. Thermodynamic process of water encapsulated in the core-shell bead

The phase diagram of water describes the thermodynamic process during heating from room temperature *T*_room_ (state 1) to the burst temperature *T*_burst_ (state 2). The whole process occurs in the subcooled liquid region. The liquid under room condition (*T*_room_, *p*_atm_) undergoes an isochoric process if it is kept heated. At the bursting temperature, the pressure *p*_burst_ is built up and reaches the critical value for the shelf. The bursting pressure is higher than the saturation pressure at *T*_burst_. Thus, if we use the saturation pressure at bursting temperature, *p*_sat@Tburst_, the value is underestimated. If we use the theoretical critical pressure to estimate the bursting temperature as the saturation temperature at this pressure, the value is over estimated. In this work, we underestimate the bursting pressure from the experimental results and overestimate the bursting temperature from the theoretical model.

S3. Normal stress inside the shell

We considered a thick-walled spherical shell subjected to a uniform internal pressure $P_{i}$ under heating. For a thick wall pressure vessel under internal pressure $P_{i}$, radial normal stress can be given by:

$\sigma_{r}$*=-*$\frac{{{(R}_{Core})}^{3}P_{i}}{r^{3}}\frac{{{(R}_{Core-Shell})}^{3}-r^{3}}{({R_{Core-Shell)}}^{3}-{(R_{Core})}^{3}}$ (4)

The inner and outer radii of the shell are denoted by $R_{Core}$ and $R_{Core-Shell}$, respectively. The maximum radial normal stress occurs at the inner surface on the plane r=$R_{Core}$: which,

${{(\sigma}_{r})}_{max}$*=-*$P_{i}$ (5)
